# Neuroimaging in schizophrenia: A review article

**DOI:** 10.3389/fnins.2022.1042814

**Published:** 2022-11-15

**Authors:** Mona Dabiri, Fatemeh Dehghani Firouzabadi, Kun Yang, Peter B. Barker, Roland R. Lee, David M. Yousem

**Affiliations:** ^1^Department of Radiology, Children’s Medical Center, Tehran University of Medical Sciences, Tehran, Iran; ^2^Department of Radiology, Boston Children’s Hospital and Harvard Medical School, Boston, MA, United States; ^3^Department of Psychiatry, Molecular Psychiatry Program, Johns Hopkins University School of Medicine, Baltimore, MD, United States; ^4^Russell H. Morgan Department of Radiology and Radiological Science, Johns Hopkins Medical Institution, Baltimore, MD, United States; ^5^Department of Radiology, UCSD/VA Medical Center, San Diego, CA, United States

**Keywords:** schizophrenia, psychosis, MRI, functional imaging, PET, DTI, psychiatry

## Abstract

In this review article we have consolidated the imaging literature of patients with schizophrenia across the full spectrum of modalities in radiology including computed tomography (CT), morphologic magnetic resonance imaging (MRI), functional magnetic resonance imaging (fMRI), magnetic resonance spectroscopy (MRS), positron emission tomography (PET), and magnetoencephalography (MEG). We look at the impact of various subtypes of schizophrenia on imaging findings and the changes that occur with medical and transcranial magnetic stimulation (TMS) therapy. Our goal was a comprehensive multimodality summary of the findings of state-of-the-art imaging in untreated and treated patients with schizophrenia. Clinical imaging in schizophrenia is used to exclude structural lesions which may produce symptoms that may mimic those of patients with schizophrenia. Nonetheless one finds global volume loss in the brains of patients with schizophrenia with associated increased cerebrospinal fluid (CSF) volume and decreased gray matter volume. These features may be influenced by the duration of disease and or medication use. For functional studies, be they fluorodeoxyglucose positron emission tomography (FDG PET), rs-fMRI, task-based fMRI, diffusion tensor imaging (DTI) or MEG there generally is hypoactivation and disconnection between brain regions. However, these findings may vary depending upon the negative or positive symptomatology manifested in the patients. MR spectroscopy generally shows low *N*-acetylaspartate from neuronal loss and low glutamine (a neuroexcitatory marker) but glutathione may be elevated, particularly in non-treatment responders. The literature in schizophrenia is difficult to evaluate because age, gender, symptomatology, comorbidities, therapy use, disease duration, substance abuse, and coexisting other psychiatric disorders have not been adequately controlled for, even in large studies and meta-analyses.

## Introduction

### Purpose and methodology

In this review article we have consolidated the imaging literature of patients with schizophrenia across the full spectrum of modalities in radiology including computed tomography (CT), morphologic magnetic resonance imaging (MRI), functional magnetic resonance imaging (fMRI), magnetic resonance spectroscopy (MRS), positron emission tomography (PET), and magnetoencephalography (MEG). We look at the impact of various subtypes of schizophrenia on imaging findings and the changes that occur with medical and transcranial magnetic stimulation (TMS) therapy. Our goal was a consolidation of the state-of-the-art imaging results in untreated and treated patients with schizophrenia.

Our review of the literature was conducted according to the preferred reporting items for systematic reviews and meta-analyses (PRISMA) guidelines with a priority placed on studies with large sample sizes, well-defined patient populations, homogeneous cohorts, and recent publication dates. Original and review articles that reported imaging findings of patients with schizophrenia were identified via searching PubMed-MEDLINE, Google Scholar, and EMBASE. Search items included schizophrenia, psychosis, MRI, CT, MRS, MEG, volumetry, diffusion tensor imaging (DTI), fMRI, treatment and TMS. We identified additional articles from the reference lists of the primary articles reviewed. We excluded (1) editorials and letters; (2) case reports or case series < 3 patients; (3) partially overlapping patient cohorts; (4) articles not written in English, and (5) non-human studies.

### Clinical aspects: Incidence of schizophrenia

Schizophrenia is a severe psychotic disorder. There are approximately 24 million patients carrying a diagnosis of schizophrenia worldwide (World Health Organization [WHO], 2022). The disease is associated with delusions, hallucinations, disorganized speech, alterations in drive and volition, impaired cognition, and mood symptoms. This disorder can make a person feel “disconnected” from reality which can be in various forms. The most common age of onset is 15–25 years for men and 25–35 years for women, and schizophrenia equally affects both genders (Häfner et al., 1994). The mean incidence of schizophrenia is 1.4–1 (male/female ratio). A late-onset variant of schizophrenia affects patients 60 years and older and is associated with social impairment (Mueller, 2022; Reichenberg, 2022).

### Clinical manifestations (according to DSM-5)

Schizophrenia can affect the physical and mental health of the patient and can disturb the person’s every-day life. This disorder includes a great range of symptoms (Table 1). Delusions are false beliefs that a person insists on regardless of valid evidence to the contrary and are frequently seen in patients with schizophrenia. Hallucinations also occur in these patients which are sensory experiences (e.g., seeing, hearing, smelling) which do not exist. Disorganized speech or speaking incoherently and unusual movements may be other symptoms that can represent the derangement of the brain. These manifestations listed above (delusions, hallucinations, speech disorders, and motor dysfunction) are sometimes referred to as “positive symptoms”. “Negative symptoms” such as a decrease in emotional expression in the face or gestures and lack of motivation (anhedonia, abulia) can also be seen in patients with schizophrenia. Other symptoms of suspiciousness (paranoia), not caring about one’s appearance and hygiene, depression, anxiety, and suicidal thoughts may manifest in these patients.

**TABLE 1:** DSM-5 criteria for schizophrenia (Malaspina et al., 2013).

• The presence of at least two of the following five items, each present for a clinically significant portion of time during a 1-month period (or less if successfully treated), with at least one of them being items (1), (2), or (3): (1) delusions, (2) hallucinations, (3) disorganized speech, (4) grossly disorganized or catatonic behavior, and (5) negative symptoms (e.g., decreased motivation and diminished expressiveness).
• For a clinically significant portion of the time since the onset of the disturbance, the level of functioning in one or more major areas (e.g., work, interpersonal relations, or self-care) is markedly below the level achieved before onset; when the onset is in childhood or adolescence, the expected level of interpersonal, academic, or occupational functioning is not achieved.
• Continuous signs of the disturbance persist for a period of at least 6 months, which must include at least 1 month of symptoms (or less if successfully treated); prodromal symptoms often precede the active phase, and residual symptoms may follow it, characterized by mild or subthreshold forms of hallucinations or delusions.
• Schizoaffective disorder and depressive or bipolar disorder with psychotic features have been ruled out because either no major depressive, manic, or mixed episodes have occurred concurrently with the active-phase symptoms or any mood episodes that have occurred during active-phase symptoms have been present for a minority of the total duration of the active and residual periods of the illness.
• The disturbance is not attributable to the physiological effects of a substance (e.g., a drug of abuse or a medication) or another medical condition. If there is a history of autism spectrum disorder or a communication disorder of childhood onset, the additional diagnosis of schizophrenia is made only if prominent delusions or hallucinations, in addition to the other required symptoms or schizophrenia, are also present for at least 1 month (or less if successfully treated).
• In addition to the symptom domain areas identified in the first diagnostic criterion, assessment of cognition, depression, and mania symptom domains is vital for distinguishing between schizophrenia and other psychotic disorders.

The DSM-4 edition described five different subtypes (paranoid, catatonic, disorganized, residual, and undifferentiated) of schizophrenia. Although they have been subsequently eliminated in the 2013 DSM-V edition because of overlap, it is useful to describe them below. These subtypes vary widely in their symptomatology, and therefore describing specific imaging patterns on the whole for schizophrenia becomes perilous. The brain function in patients with paranoid schizophrenia manifested by frequent hallucinations, delusions, psychotic behavior, and disorganized speech and thinking is unlikely to be the same as in patients in catatonia where the symptoms may be mutism, echolalia, and abulia, coupled with absent (or excessive) voluntary movements. Disorganized schizophrenia occurs in the absence of hallucinations and delusions but is largely manifested by incomprehensible expressive speech. The patient may have a flat affect and disorganized thinking. Residual schizophrenia refers to the symptom complex when the patient has recovered from a psychotic break or delusions but still may be emotionless and disorganized in speech and thought. If a patient has a combination of the behaviors described above and does not fit neatly into one subtype, the patient’s condition is termed undifferentiated.

The following disorders should be considered when making a clinical diagnosis of schizophrenia. One of the biggest challenges is to discern between schizophrenia and bipolar disorder (manic-depressive disease) since 50% of manic episodes in patients with manic-depressive disorder may mimic the positive symptoms of schizophrenia (these include grandiose delusions, hallucinations, slurred speech, paranoia, psychosis, etc.) The negative signs of schizophrenia can resemble depressive episodes of bipolar disorders or major depressive disorder (these include apathy, extreme emotional withdrawal, lack of affect, low energy, social isolation, etc.) (Greene et al., 2018).

### Risk factors

There are no specific and confirmed causes of schizophrenia. However, there are some risk factors attributable to this disorder. Genetic risk (specifically parents or siblings with schizophrenia) is considered one of the important predisposing factors. Environmental factors such as being born in winter, living in an urban environment, air pollution (PM_2_._5_, PM_*C*_, PM_10_, SO_2_, and NO_2_ exposure) (Song et al., 2022), immigrating to a new country, using cannabis or recreational drugs, especially from a young age, and suffering prenatal hypoxic ischemic events and/or nutritional deficiencies (Susser et al., 1995), constitute risk factors (Tiwari et al., 2010). Certain infections (e.g., influenza, toxoplasma gondii, herpes simplex virus type 2) (Brown and Derkits, 2010) and autoimmune diseases can increase the risk. Suffering from a severe long-term stress has been demonstrated to affect the brain and raise the risk of schizophrenia. Birth factors such as (1) maternal gestational diabetes, (2) preeclampsia, (3) *in utero* malnutrition and vitamin D deficiency, (4) twin gestation, (5) delivery by cesarean section, (6) history of asphyxia (Wortinger et al., 2022), and (7) underweight at birth have been described as additional correlations (Davies et al., 2020).

### Prognosis

There is no certain cure for schizophrenia, however, the symptoms of schizophrenia can be treatable. A small percentage of patients can recover entirely. Nearly one-third of people with schizophrenia will positively respond to pharmaceutical and behavior modification treatment and they can have a normal or mostly normal life. The disorder is not fatal itself but it can lead to behaviors that can be harmful to the patients and people around them. Nearly 10% of patients with schizophrenia die from suicide. They also may develop chronic conditions such as heart disease. Worse outcomes are associated with a family history of psychosis, substance use, earlier onset of disease, and longer duration of untreated psychosis (Andreasen et al., 2005).

Therefore, due to myriads of risks that people with schizophrenia are dealing with, it is crucial to detect and treat schizophrenia in a timely manner (Waszczuk et al., 2021).

## Imaging

### Why imaging is important

The main role of imaging, as with most psychiatric disorders, is to exclude structural lesions in the brain that may lead to symptoms that simulate schizophrenia. For example, anterior cranial fossa meningiomas that encroach on the frontal lobes are known to cause such symptoms as abulia, personality changes, and olfactory hallucinations which may lead to a misdiagnosis of schizophrenia. Standard CT and MR imaging are unable to “diagnose” schizophrenia, but the exclusion of other lesions is an important component of the work-up of patients with psychotic symptoms.

### Standard computed tomography

Conventional imaging of the patient with an acute psychotic episode who is seen in the Emergency Department often starts with a head CT. Although there are no signature features of schizophrenia on CT scanning, this initial evaluation is used to identify other brain lesions that may lead to symptoms mimicking schizophrenia.

Computed tomography scans may show “volume loss greater than expected for age” in the frontal lobes, temporal lobes, caudate heads, and thalami of patients with schizophrenia (Ellison-Wright et al., 2008; Torres et al., 2016). This volume loss progresses over the long-term course of the disease. However, some studies suggest that these changes may be a byproduct of the long-term medications used to treat the disease and/or comorbidities, rather than the disease itself. In one study, serial CT scans were evaluated in 43 patients over a mean of 13.0 years of follow-up and the expansion of the subarachnoid space correlated strongly with the cumulative dosage of anti-psychotic medications (Kanahara et al., 2022). In another study, 43 patients with relapsing episodes of psychosis were evaluated over 13 years. The authors found a positive relationship between expansion of cerebrospinal fluid (CSF) space and antipsychotic drug dosage. Patients who were treated with typical antipsychotics had a greater brain volume reduction as opposed to ones who were just treated with atypical antipsychotics (Kanahara et al., 2022).

Although CT is often the first study performed in new onset psychosis, MRI and functional imaging have proved the mainstay for schizophrenia assessment in both the clinical and research realms.

### Standard magnetic resonance imaging

**FIGURE 1:**
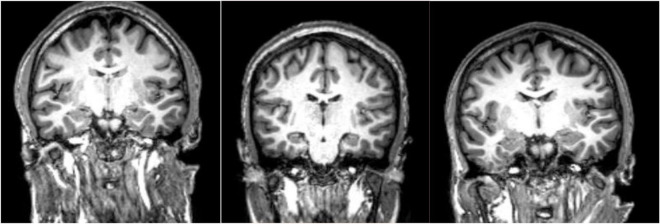
MPRAGE images of three patients with schizophrenia. These type of high resolution T1-weighted images allow quantitation of specific gray matter and cerebrospinal fluid (CSF) containing structures which has led to the conclusion that patients with schizophrenia have overall decreased gray matter volumes and enlarged CSF spaces. Specific cortical regions are best evaluated in group analyses and/or voxel-based morphometry analyses. Comparing these three subjects, one finds reduced right versus left hippocampal volume in the second and third subjects at group level, a finding that is not necessarily confirmed by visual analysis at individual level.

Due to the high resolution of MRI, various brain abnormalities in schizophrenia such as increased volume of cerebrospinal fluid (CSF) and decreased volume of white and gray matter have been detected (Sadeghi et al., 2022; Figure 1). Quantitative analyses of rigorous volumetric data have identified volume loss in patients with schizophrenia in the gray matter of thalamocortical connections and the prefrontal cortex (Kuo and Pogue-Geile, 2019; Radua et al., 2020). Histologically this gray matter reduction is accompanied by dendritic and synaptic density decreases which likely signals a lack of communication (disconnection theory) across selected neural networks (Glantz and Lewis, 2000).

In a meta-analysis which included 4,789 patients with schizophrenia evaluated with voxel based morphometry, Picó-Pérez et al. (2022) noted reductions in gray matter volume in the following areas of the brain: inferior, middle and superior frontal gyri, the inferior, middle and superior temporal gyri, the parahippocampal gyri, the posterior and anterior insulae, the medial prefrontal cortex, dorsal anterior cingulate cortex, posterior cingulate cortex, bilateral angular and supramarginal gyri, bilateral thalami and caudate nuclei. However, a second meta-analysis noted progressive volume loss from high-risk subjects to recently diagnosed schizophrenia to patients with chronic schizophrenia most strongly in the right anterior cingulate and mesial temporal lobe structures (cortico-striatal-limbic hub regions) (Liloia et al., 2021). Another group also found that patients who have schizophrenia with psychosis had larger Sylvian fissures and cingulum sulci (*p* = 0.027) than healthy controls. Concomitant volume loss in the planum temporalis, insula, and cingulum was implied, a characteristic feature of the brains in patients with psychosis (Lee et al., 2016; Faria et al., 2021).

No studies have found any areas of increased gray matter volume in patients with schizophrenia.

Many groups have combined volumetric studies with other modalities to assess this disease. The group led by Akira Sawa utilized a multimodal longitudinal cohort to investigate biomarkers for treatment-resistant psychosis. Their study observed reductions in hippocampal volumes and glutathione (GSH) levels in the anterior cingulate cortex in treatment resistent patients compared to responsive patients (Yang et al., 2022). Interestingly, they found that a combination of multimodal biomarkers could lead to a better prediction of treatment resistance than any individual biomarker. Such research efforts showed that comprehensive studies of multimodal longitudinal data could be useful for identifying biomarkers for schizophrenia or even subsets of patients with schizophrenia.

### Functional imaging

#### Positron emission tomography

##### FDG Positron emission tomography

The idea that people with schizophrenia may have a different pattern of cerebral glucose utilization from healthy volunteers was first suggested by Buchsbaum et al. (1982). Many researchers have noticed “hypofrontality” in fluorodeoxyglucose positron emission tomography (FDG PET) investigations of schizophrenia, which implies a decreased frontal to occipital lobe metabolic ratio of FDG utilization (Farkas et al., 1984; Wolkin et al., 1985). The frontal and temporal cortex of the patients showed decreased metabolism, but not the parietal and occipital lobes (Buchsbaum et al., 2007). In a recent study using PET to compare between schizophrenia and narcolepsy, Chin et al. confirmed hypometabolism in the frontal region in the schizophrenia group (Baron et al., 2013). In addition, a recent systematic review and meta-analysis confirmed the hypotheses of hypofrontality in schizophrenia (Whitehurst et al., 2020; Townsend et al., 2022). However, not all investigations were able to repeat the same result (Sheppard et al., 1983; Whitehurst et al., 2020) and one study even discovered hyperfrontality (Soyka et al., 2005). According to a meta-analysis by Whitehurst et al. (2020) while reductions in *N*-acetyl aspartate levels may occur in chronic schizophrenia in the temporal lobe, parietal lobe, and hippocampus, they are localized to the frontal lobe in early psychotic illness. In this case *N*-acetyl aspartate levels corroborated FDG PET results as a marker of neuronal metabolic function and therefore the hypofrontality theorem. Another theory is that hypometabolism results from aberrant synaptic function, which leads to decreased demand. A significant body of data links synaptic dysfunction to the pathophysiology of schizophrenia, including *in vivo* and post-mortem findings of reduced frontal synaptic protein levels (Onwordi et al., 2020). Thus, neuronal glucose demand may decrease when synaptic activity declines. Further research is required to examine whether glucose metabolic abnormalities are related to mitochondrial or synaptic dysfunction in schizophrenia.

There are FDG PET distinctions in the two types of schizophrenia’s clinical manifestations regarding positive symptoms (delusions and hallucinations) versus negative/deficit symptoms (abulia, withdrawal). The negative symptom form of schizophrenia is linked to lower metabolism in all regions of interest in cortical and subcortical structures on PET. Patients with the deficit type of schizophrenia had lower glucose absorption than patients with the non-deficit type in all neocortical regions of the brain (frontal, parietal, temporal, and occipital), as well as the thalamus and the limbic system (Tamminga et al., 1992). In positive symptom schizophrenia, dysfunctions in the temporolimbic system have been identified. A substantial increase in maximum glucose uptake can be seen in the left thalamus, basal ganglia, and medial temporal areas with higher positive syndrome scores (Bogerts, 1997).

As far as other variables that may affect the FDG PET results, Buchsbaum et al. (1982) noted that decreased cerebral glucose uptake first develops early in the course of a disease and is unrelated to chronicity, the intensity of the patient’s symptoms, or drug exposure. In contrast, Gur et al. (1987) and Szechtman et al. (1988) found that brain metabolism is influenced by the severity and length of the illness. However, in a study by Seethalakshmi et al. (2006) neither the severity of the disease nor its duration had any bearing on the uptake of glucose by the brain. These factors (effect of disease duration and severity of symptoms) are an ongoing subject of debate but may be explained by the positive-negative symptom uptake discrepancy which has not been controlled for across these studies.

##### Other tracers

A meta-analysis of non-FDG tracers used to evaluate patients with schizophrenia has recently concluded that a functional excess of brain dopamine in schizophrenia has been supported by many studies. Many different agents (FDOPA, DAT ligand [11C]PE2I, SPECT studies) show higher than normal uptake of the dopaminergic agonists in the striatal basal ganglia (caudate, globus pallidus, and putamen) of unmedicated patients. The early finding of increased dopamine synthesis capacity, however, is not pathognomonic of schizophrenia, since nearly half of patients have normal dopamine synthesis capacity and other patients with psychiatric manifestations may have elevated FDOPA accumulation as well. Once patients with schizophrenia are on antipsychotic medications, there may be normalization of these dopamine uptake studies (Cumming et al., 2021).

Cannabinoid receptor binding PET agents have been applied to patients with schizophrenia based on the observation that cannabis may be associated with acute psychoses and its use appears to be a risk factor for schizophrenia. In a study of 67 patients with schizophrenia, increased cannabinoid receptor availability was observed using a [^18^F]MK-9470 agent in the nucleus accumbens, insula, cingulate cortex, inferior frontal gyrus, parietal and mesotemporal lobe regions of the brain (Ceccarini et al., 2013). The higher the cannabinoid receptor availability in the nucleus accumbens the lower the incidence of negative symptoms of schizophrenia in the patients.

Most serotonin binding PET agents have failed to detect a reproducible increase or decrease in uptake in schizophrenia, despite many different agents being employed (Cumming et al., 2021).

**FIGURE 2:**
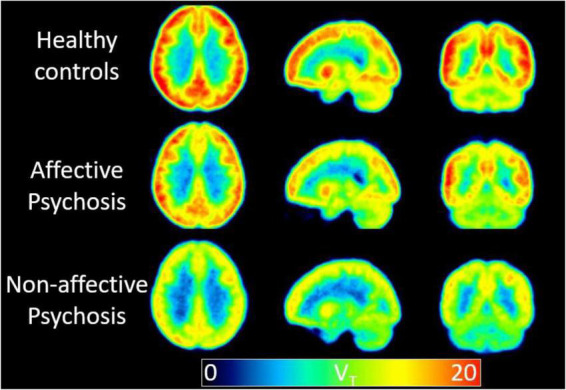
A low availability of the alpha7 nicotinic acetylcholine receptor in non-affective psychosis is supported by the binding (V_T_) values using ^18^F-ASEM PET. Mean parametric ^18^F-ASEM V_T_ images derived from a study population of 15 controls (top), six patients with affective psychosis [Bipolar I disorder (middle)], and five patients with non-affective psychosis [Schizophrenia, Schizoaffective disorder (bottom)]. Images from left to right: axial, sagittal, and coronal views. V_T_ is in reported in mL⋅cm^–3^ (Coughlin et al., 2018).

A study by Wong et al. (2018) showed a reduced hippocampus and cingulate availability of the α7 nicotinic acetylcholine receptor (α7-nAChR) in non-smokers with recent-onset psychosis, especially those with non-affective (NP) psychosis, and its connection with cognitive deficits after correcting for age. Additionally, Olincy, by using 18F-ASEM (a PET ligand directed to this α7-nAChR receptor) showed that the median volumes of distribution values in the cingulate and frontal cortices and hippocampus were lower in patients than in age-matched healthy volunteers (Wong et al., 2018). Another group, however, showed lower 18F-ASEM binding in the hippocampus of patients with recent onset psychosis compared to controls (Figure 2). This observation correlated with lower cognitive performance (Coughlin et al., 2018).

#### Functional magnetic resonance imaging

##### Perfusion imaging

Arterial spin labeled (ASL) MRI measures cerebral blood flow directly by inverting the magnetization of arterial blood water using radiofrequency pulses to create an endogenous diffusible tracer (Alsop et al., 2015), in contrast to fMRI, which measures neuronal activity indirectly through changes in blood flow and oxygen metabolism (Hoptman et al., 2022). Studies using arterial spin labeling to evaluate patients with schizophrenia are typically small and have had variable results (Théberge, 2008; Guimarães et al., 2016). Nevertheless, frontal hypoperfusion during rest has been documented in multiple studies (Scheef et al., 2010; Pinkham et al., 2011; Walther et al., 2011; Ota et al., 2014; Kindler et al., 2015, 2018; Zhu et al., 2015), with the anterior cingulate cortex (Scheef et al., 2010; Kindler et al., 2015; Zhu et al., 2015) and the medial frontal gyrus (Pinkham et al., 2011; Walther et al., 2011; Kindler et al., 2015; Zhu et al., 2015) being the most frequently studied regions. By contrast, higher relative cerebral blood flow levels in the thalamus were observed in non-medicated patients (Scheef et al., 2010) in comparison to healthy controls in a major investigation that compared 106 chronic schizophrenia individuals to 94 healthy controls. Additionally, in this large sample size study, larger relative cerebral blood flow levels were also discovered in the putamen and inferior temporal gyrus, while decreased relative cerebral blood flow levels were discovered in the frontal and occipital regions and remained unchanged in the caudate nucleus (Zhu et al., 2015). This literature has not been definitive since two smaller studies (Walther et al., 2011; Liu et al., 2012) indicated reduced thalamic blood flow measured using ASL, and both higher (Liu et al., 2012) and lower (Kindler et al., 2015) relative cerebral blood flow were found in the caudate nucleus. Unaltered or reduced relative cerebral blood flow has been recorded in the parietal lobes. Lower relative cerebral blood flow in the parietal lobe was confirmed in two other studies (Platek et al., 2006; Hodzic et al., 2009).

Wright et al. (2015) used diffusion weighted images and ASL to relate cognitive deficits in patients with schizophrenia with their brain metabolic rate and microstructural status. They show that the reduced speed of information processing in schizophrenia patients is explained by reduced whole brain white matter integrity, mediated by a mismatch in white-gray matter blood perfusion observed through ASL. This interaction between brain perfusion and white matter integrity, and its relation with processing speed, was latter observed in the corpus callosum of individuals with early schizophrenia, and was independent of the duration of the illness and medication dose (Castro-de-Araujo et al., 2018).

##### Task-based functional magnetic resonance imaging

Blood oxygenation level-dependent (BOLD) fMRI is a method that relies on deoxyhemoglobin’s T2 shortening magnetic susceptibility effects, which result in regional signal intensity alterations on susceptibility-sensitive imaging sequences (e.g., echoplanar or routine gradient echo sequences). Signal intensity is seen to rise overall in areas of the brain that are stimulated by task demands. This is due to an increase in regional oxygenated blood flow that is greater than the region’s oxygen consumption resulting in a decrease in the level of deoxyhemoglobin and thus a higher signal intensity on T2* scans. Event/Task-related fMRI uses the BOLD phenomenon to contrast the signal during stimulus presentation or performance of a task with that of a control stimulus or non-task.

When different task-based paradigms have been tested across the domains of working memory, cognitive problem solving, and interpersonal communications, patients with schizophrenia have consistently shown decreased activation in the prefrontal cortex. One working memory task study showed that, with practice (which normally leads to smaller volumes of activation), medication naïve patients with schizophrenia showed a smaller reduction in activation versus controls in the dorsolateral prefrontal cortex which correlated with the severity of negative symptoms in patients (van Veelen et al., 2010). When the tasks have interrogated social/emotional situations the right occipital, right fusiform, amygdala, hippocampus, and anterior cingulate cortex have demonstrated hypoactivation compared with controls (Li et al., 2010; Anticevic et al., 2012; Taylor et al., 2012).

A recent meta-analysis by Pico-Perez et al. looked at 4,789 patients’ structural and functional studies across the domains of processing speed, attention/vigilance, working memory, verbal learning and memory, visual learning and memory, executive function/reasoning, problem solving, verbal fluency, and verbal comprehension, emotional perception, social perception and knowledge, theory of mind, and attributional bias. The authors noted dorsomedial prefrontal cortex, supplementary motor area, and right inferior frontal gyrus decreases in activation when presented with cognitive tasks (data from 3,446 patients). These authors noted that, through their meta-analysis, they were able to isolate the attention/vigilance tasks as the ones that most uniformly showed deficits in patients with schizophrenia across multiple studies. This comports with some of the DTI and resting state fMRI studies that highlight the salience network (see below). However, when the tasks were more social interaction/skills based, the reduction in brain activation was more focused in the right angular gyrus (1,755 patients with schizophrenia) (Picó-Pérez et al., 2022). No areas of hyperactivation in either of these task-based scenarios was observed. Another meta-analysis showed overlapping task and resting-state fMRI abnormalities in the prefrontal regions, including the dorsal lateral prefrontal cortex, the orbital frontal cortex and the temporal lobe, especially in the left superior temporal gyrus (Mwansisya et al., 2017).

One study looked at 142 patients with schizophrenia having auditory verbal hallucinations who underwent task-based fMRI. The authors discovered that fMRI activation appeared to localize to the left insula and inferior parietal lobule during the auditory verbal hallucinations and to be associated with significantly reduced gray matter volume.

When the Stroop task (saying a color of a word when it is printed in the correct or non-matching color) was compared between 42 patients with schizophrenia and 61 control subjects, the authors found deactivations in the medial frontal cortex, the middle and posterior cingulate gyrus and cuneus, the parahippocampal gyrus and the hippocampus in healthy controls. However, the patients with schizophrenia showed failure of deactivation in the medial frontal cortex suggesting default mode network (see below) dysfunction in schizophrenia (Salgado-Pineda et al., 2021). When negative threat words were used in a task-based fMRI experiment, the angular gyrus, middle/inferior temporal gyrus and the amygdala were hyperactivated in patients with schizophrenia compared to controls. The more excitement/hostility elicited by these words, the greater the left dorsal temporal pole and dorsal anterior cingulate activation in patients with schizophrenia. This was the only large study that found hyperactivation in patients with schizophrenia but most studies do not control for positive/negative symptom dominance among the subjects, a source of possible discrepancies (Dar et al., 2021).

##### Resting state functional magnetic resonance imaging

**FIGURE 3:**
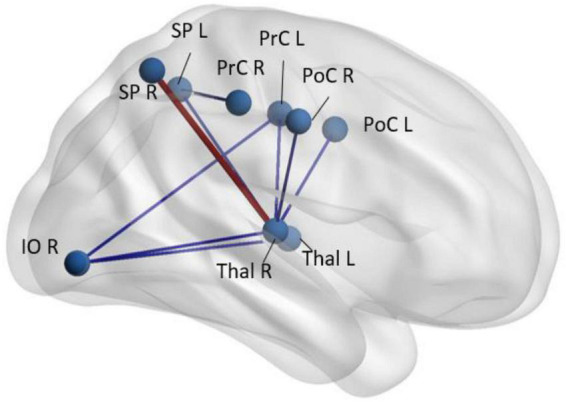
Regions with abnormal synchrony in rs-fMRI time-courses in early-schizophrenia patients (*n* = 58) compared to age- and sex-paired healthy controls. Blue codes decreased and red codes increased pairwise rs-fMRI correlations in schizophrenia patients. SP, superior parietal; Thal, thalamus; PrC, pre-central gyrus; PoC, post central gyrus; IO, inferior orbital; R, right; L, left (Faria et al., 2021).

Resting-state fMRI (rs-fMRI) has been extensively employed in evaluating patients with schizophrenia but, in addition to the patient factors described above which are not controlled for, the studies are plagued by multiple methodological differences, variable analysis techniques used, and small sample sizes (Zhang et al., 2009). rs-fMRI can interrogate multiple neural networks in the brain simultaneously, even without stimuli or task (Figure 3). The basis of rs-fMRI is on synchronies of low frequency fluctuations (less than 0.1 Hz) in the BOLD signal (Lee et al., 2013). Using resting-state fMRI, multiple brain networks have been discovered, including the default mode network, central executive network, salience network, language, sensorimotor, auditory and visual networks (Raichle et al., 2001; Fox et al., 2005; Seeley et al., 2007; Bressler and Menon, 2010; Cabral et al., 2014). The networks that have been studied most extensively with rs-fMRI that have been implicated in schizophrenia have been the default mode network and salience network (Sridharan et al., 2008; Bressler and Menon, 2010; Menon and Uddin, 2010; Palaniyappan and Liddle, 2012).

The frontal-dorsal medial prefrontal cortex, ventral medial prefrontal cortex, posterior medial cortex, and posterior lateral cortex make up the majority of the default mode network (Palaniyappan and Liddle, 2012). There have been reports of default mode network anomalies in schizophrenia in several research studies (Bluhm et al., 2007; Zhou et al., 2007; Whitfield-Gabrieli et al., 2009; Mannell et al., 2010; Camchong et al., 2011; Jang et al., 2011; Mingoia et al., 2012; Fan et al., 2022), and other studies have looked at an association between the symptoms of schizophrenia and abnormal functional activity in default mode network brain areas (Garrity et al., 2007; Zhou et al., 2007; Jang et al., 2011; Mingoia et al., 2012). According to some data, the strength of positive symptoms is correlated with the default mode network, central executive, and salience networks’ dysconnectivity (Rotarska-Jagiela et al., 2010; Woodward et al., 2011; Khadka et al., 2013; Palaniyappan et al., 2013). The failure to effectively allocate resources between internal thoughts and external inputs may be linked to abnormal connections in the prefrontal cortex (Jang et al., 2011). Aberrant default mode network function in schizophrenia may be the cause of mental abnormalities including delusions, which are closely related to spontaneous ideas (Mason et al., 2007). The recognition of internal and exterior stimuli mediated through the salience network) and the transition of brain activity from default mode network to central executive network activities have been attributed to the salience network (Sridharan et al., 2008; Palaniyappan and Liddle, 2012). Deficits in salience network may be associated with hallucinations, delusions, disarray, and psychomotor underdevelopment in schizophrenia (White et al., 2010; Palaniyappan et al., 2011a; Palaniyappan and Liddle, 2012; Pu et al., 2012).

Overall symptom burden was greater in patients with smaller default mode network and salience network activation surface areas, according to Palaniyappan et al. (2011b), who also found that schizophrenia patients had reduced surface area across all three intrinsic networks (default mode network, salience network, and central executive network). According to Orliac et al. (2013), patients with schizophrenia have reduced functional connectivity in the default mode network and salience network. Furthermore, decreased connectivity in the paracingulate cortex is associated with difficulties with abstract thought, whereas decreased connectivity in the left striatum is associated with delusions and depression. Longer memory response time for face recognition was also associated with functional connectivity abnormalities in early-schizophrenia, centered in the anterior cingulate (Narita et al., 2021). The default mode network (and striatal) network dysfunction has also been used to predict treatment response to antipsychotic medications (Mehta et al., 2021).

The connections of the thalamocortical network have also been implicated in schizophrenia and psychosis with reduced connectivity between the mediodorsal thalamus and the insula and orbitofrontal regions (Ramsay et al., 2022). Greater symptomatology and worse cognitive deficits were associated with reduced thalamo-insular connectivity. Such deficits in mediodorsal thalamic connectivity to frontal regions were duplicated in patients with schizophrenia with auditory verbal hallucinations (Wei et al., 2022) while others have noted interhemispheric connectivity deficits in temporal lobe regions in the same population (Chen et al., 2022). Another resting-state fMRI study supported the theory that the thalamus-related network disconnection, specifically with the bilateral postcentral and superior temporal gyri, temporal pole, inferior lateral occipital cortex, and vestibulo-cerebellum. They stated the disconnection occurs in early stages of schizophrenia and can predict negative, positive, and cognitive symptoms of the disease (Chen et al., 2020). Abnormalities in rs-fMRI signal centered in the thalamus were independently observed in patients with early-stage schizophrenia (Faria et al., 2021). These findings also tie back to the volumetric studies cited above in which volume loss in the gray matter of thalamocortical connections and the prefrontal cortex has been reported in patients with schizophrenia (Kuo and Pogue-Geile, 2019; Radua et al., 2020).

Using the same ROI pairings in the same anatomical area, Hoptman et al. evaluated the connection between resting state functional connectivity and structural connectivity in the default mode network in patients with schizophrenia and healthy controls. Data from the DTI and resting state fMRI sequences did not generally correlate, however, connections between the prefrontal cortex, posterior cingulate, and lateral temporal lobes with frontal and parietal regions did correlate (Szechtman et al., 1988). This is in line with the frontotemporoparietal network disruption theory in schizophrenia that is well-known (Friston and Frith, 1995).

In a study that conducted by Lottman et al., patients with schizophrenia showed an increased connectivity between auditory and subcortical networks. Also, a decrease connectivity between interhemispheric homotopic sensorimotor networks in rs-fMRI were seen in these patients (Lottman et al., 2019).

A typical metric in rs-fMRI called regional homogeneity defines the local synchronization of blood oxygenation level-dependent signals by comparing the time series of a particular voxel to the closest voxel (Zang et al., 2004). The regional homogeneity method is a productive, repeatable, and reliable neuroimaging mark that identifies local functional connectivity (Zuo et al., 2013). Using this method, studies have shown schizophrenia is characterized by broad regional homogeneity abnormalities. For instance, people with schizophrenia displayed greater regional inhomogeneity values in the dorsolateral prefrontal cortex, visual areas, and basal ganglia networks (including caudate, putamen, and globus pallidus) (Liu et al., 2019).

Furthermore, patients with schizophrenia showed elevated regional homogeneity values in the prefrontal cortex (Shan et al., 2021), inferior parietal lobule, precuneus/cuneus (Zhao et al., 2019), cerebellum, as well as decreased regional homogeneity values in the temporal lobule, medial prefrontal gurus (Shan et al., 2021), inferior parietal lobule (Zhao et al., 2018), precuneus (Yan et al., 2020), and occipital lobule (Gao et al., 2018). Regional homogeneity and cognitive dysfunction were recently linked in a large study by Huang et al. (2022) of first-episode schizophrenia patients. They showed that in patients with schizophrenia, regional homogeneity values were increased in the right superior frontal gyrus and decreased in the left anterior cingulate gyrus, right middle occipital gyrus, left cuneus, and right superior occipital gyrus.

#### Diffusion tensor imaging

Diffusion tensor imaging (DTI) has paved the way for investigating the white matter tracts in the brain *in vivo* in health and disease. A large-scale study from the ENIGMA group identified widespread white matter microstructural abnormalities in chronic schizophrenia (Kelly et al., 2018). These widespread DTI abnormalities were later observed in early-schizophrenia patients. Other DTI studies have focused on the tracts leading to and from the amygdala as one of the cerebral structures affecting behavior and mood. The uncinate fasciculus is of particular interest since it connects the mood region (amygdala) with the executive function regions (the medial- and orbitofrontal cortices). Reduced fractional anisotropy is seen in patients with schizophrenia suggesting diminished white matter structural integrity (Ho et al., 2019). In another study the apparent diffusion coefficient (ADC) and radial diffusivity (RD) showed elevations in patients with schizophrenia in the corpus callosum and cingulum (Nenadić et al., 2017). Fractional anisotropy values were reduced across all regions of the corpus callosum although similar findings are found in bipolar patients (Li et al., 2014). When patients at risk for schizophrenia (based on various surveys and examinations) were divided into those that progressed to schizophrenia versus those that did not, one group found more severe fractional anisotropy and radial diffusivity changes in the corpus callosum in at risk subjects who progressed to schizophrenia versus those who did not progress (Smigielski et al., 2022).

There may be a difference between treatment responsive and non-responsive patients as far as their DTI findings. This ties in to the relationship between the effectiveness of antipsychotic drugs and their affinity for dopamine receptors, particularly in the striatum where dopamine synthesis is elevated in schizophrenia (see “Positron emission tomography” section above). The associative striatum which receives afferent connections from the dorsolateral prefrontal cortex may play a role in psychosis by striatal dopaminergic dysregulation. To that end, one group has found an inverse correlation on DTI studies between the connectivity of the dorsolateral prefrontal cortex-associative striatum and dopamine synthesis in treatment responsive patients with schizophrenia, which is not present in treatment resistant patients (Shin et al., 2022). The disconnection of the frontostriatal network is worse in treatment sensitive patients.

A recent review of 401 studies looking at the white matter in schizophrenia reported that the vast majority of authoritative papers showed decreased fractional anisotropy, predominantly in the corpus callosum (Jelsness-Jørgensen et al., 2012). However, the longitudinal fasciculi were also frequently affected with lower fractional anisotropy (right > left) as were regions in the cingulum, left > right (Zhao et al., 2022). As far as specificity, four of the neuropsychiatric disorders studied, obsessive-compulsive disorder, major depressive disorder, bipolar disorder, and schizophrenia had abnormal DTI measures in the corpus callosum and the superior longitudinal fasciculus, to varying degrees (schizophrenia the worst) (Luttenbacher et al., 2022). As far as the impact on the patients, decreased fractional anisotropy has been linked to the deficits in patients with schizophrenia in both positive and negative symptoms, as well as executive, memory, and social dysfunction (Karlsgodt, 2020).

**FIGURE 4:**
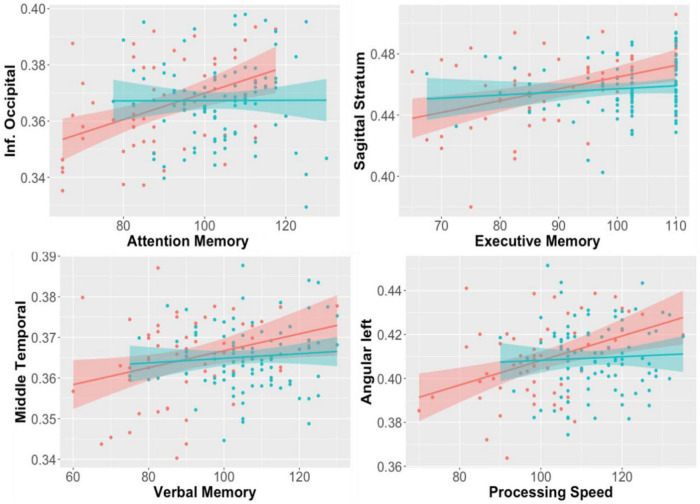
Correlations between regional fractional anisotropy (FA) in diverse brain areas (Y axis) and performance in cognitive tests (X-axis). Red dots are early-stage schizophrenia patients (*n* = 58); blue is age- and sex-paired healthy controls. The shadow represents the 95% interval for the linear fitting (line). Significant correlations between FA and cognition were found in the schizophrenia group, but not in controls (Faria et al., 2019).

**FIGURE 5:**
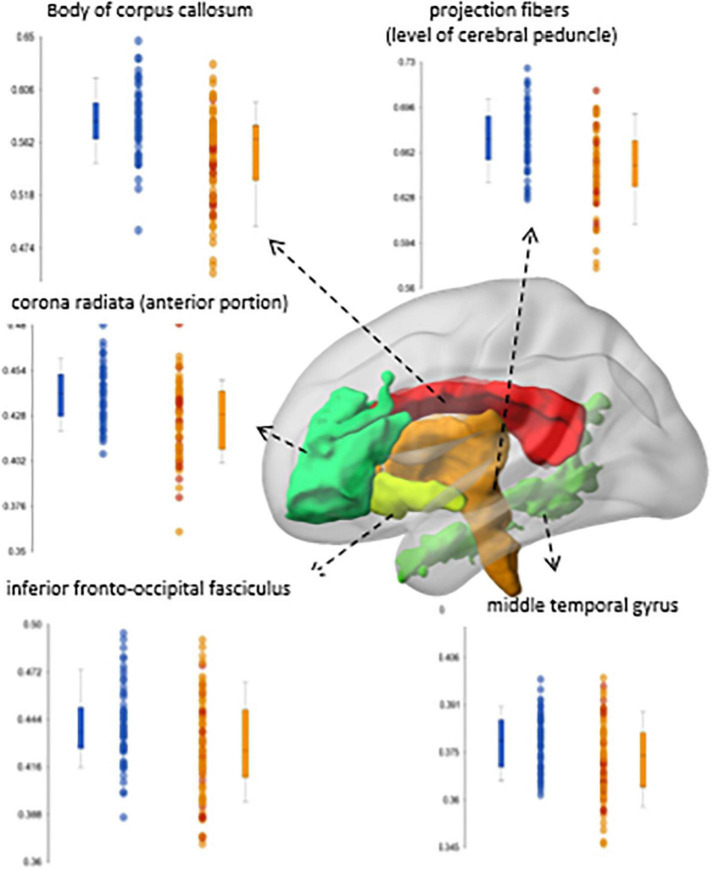
Significant differences in fractional anisotropy (FA) between a group of early-stage schizophrenia patients (*n* = 58; orange dots in the box-plots) and age- and sex-paired healthy controls (blue dots in the box-plots) (Faria et al., 2021).

In patients with first episode psychosis, white matter anisotropy DTI studies showed whole brain decreases in fractional anisotropy compared to controls (Faria et al., 2019, 2021); this diminution correlated with decreases in cognitive function of the subjects (Figure 4). Projection fibers, commissural fibers (i.e., corpus callosum) and association fibers show lower fractional anisotropy, but there can be higher fractional anisotropy in the caudate nucleus. Processing speed seemed to be most closely correlated with DTI measures in the temporal lobe gyri. These correlations are strongest in patients with schizophrenia with psychosis, compared to manic depressive patients with psychosis (Faria et al., 2019). In patients with first episode psychosis, DTI and resting state fMRI abnormalities were found centered in the thalamus (Faria et al., 2021), harkening back to volumetric and fMRI studies which also support the thalamocortical connection dysfunction theory (Figure 5).

#### Magnetic resonance spectroscopy

**FIGURE 6:**
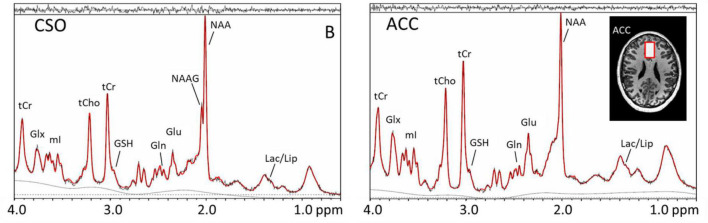
Magnetic resonance (MR) spectra recorded at 7T in the centrum semiovale (CSO) white matter and anterior cingulate cortex (anterior cingulate cortex) using the STEAM pulse sequence and VAPOR water suppression in a xx-year-old subject with schizophrenia. Voxel locations and sizes are indicated overlaid on T1-weighted MR images. In the spectra, peaks are detected from lactate (Lac), lipid (Lip), *N*-acetyl aspartate (*N*-acetylaspartate), *N*-acetyl aspartylglutamate (*N*-acetylaspartateG, predominantly in white matter), glutamate (Glu), glutamine (Gln), glutathione (GSH), total creatine (tCr), total choline (tCho), myo-inositol (mI), and a combined glutamate and glutamine peak (Glx). While these spectra are virtually indistinguishable from those observed in healthy control subjects, quantitative analysis and studies in sufficient numbers of subjects reveal a number of statistically significant differences between patients and controls.

Magnetic resonance spectroscopy (MRS) is a non-invasive technique that has been used extensively for the evaluation of brain metabolism in neurological and psychiatric disorders, such as schizophrenia. MRS can be performed using several different MR-active nuclei, including ^1^H, ^31^P, and ^13^C. Among them, ^1^H is the most sensitive and by far the most commonly used since no additional hardware is required to perform it on clinical MRI scanners (unlike the other nuclei). When performed at high magnetic field strengths (such as 7T) nearly 20 neurochemicals can be measured in the human. These include neurotransmitters/modulators such as glutamate, glutamine, gamma aminobutyric acid, aspartate and *N*-acetylaspartyl asparate glutamate (Duarte and Xin, 2019). Other compounds which can be detected include neuronal *N*-acetylaspartate and glial cell (choline and myo-inositol) “markers,” compounds related to glycolysis (lactate), as well as antioxidants (glutathione and ascorbate) (Figure 6). Glutamate is the most abundant excitatory neurotransmitter in the brain which may be involved in many neuropsychological disorders. Abnormal levels of glutamate and glutamine have been reported in schizophrenia, indicating possible glutamatergic dysfunction, hypofunction of *N*-methyl-D aspartate receptors, and/or metabolic impairment at the mitochondrial level (Duarte and Xin, 2019).

With 7 Tesla MRS, one can derive high resolution quantitative neurochemical profiles. In schizophrenia, lower glutamate in the anterior cingulate cortex and higher levels in the centrum semiovale were noted at 7T (Andrade et al., 2016). Others have found decreases over time in gamma aminobutyric acid, *N*-acetylaspartate, *myo*-inositol, and total choline in the anterior cingulate cortex in patients with first episode psychosis patients (Wang et al., 2020). Longer illness duration group had higher levels of lactate compared to the shorter illness duration group in the centrum semiovale. Lower levels of glutamate, *N*-acetylaspartate and glutathione in first episode psychosis patients were seen in five locations in the brain. The lower levels of *N*-acetylaspartate in the centrum semiovale correlated with worse performance in neuropsychological tests in the patients with FEP. Lower levels of glutamate and glutathione in the anterior cingulate cortex of patients with schizophrenia at 7T have also been reported. In general, the most consistent finding across most studies is decreased *N*-acetylaspartate in schizophrenia and first episode psychosis which suggests the likelihood of neuronal damage/dysfunction even early in the disease course.

Metabolic changes in the dorsolateral prefrontal cortex and the visual cortex in schizophrenia were evaluated on 40 patients. They found a high level of choline in dorsolateral prefrontal cortex and the visual cortex. However, glutamate, creatine, mayo-inositol, and *N*-acetylaspartate levels showed no changes in these regions. They also discovered that there is a negative correlation between *N*-acetylaspartate levels in dorsolateral prefrontal cortex and negative symptoms in schizophrenia (Smucny et al., 2022).

Low medial frontal cortex glutamate levels were confirmed in a meta-analysis of 1,251 patients with schizophrenia compared with 1,197 healthy volunteers (Merritt et al., 2021). A higher glutamate to creatine ratio in the medial frontal cortex was (1) positively associated with overall symptom severity, (2) positively associated with positive schizophrenia symptomatology severity, and (3) negatively associated with global functioning.

**FIGURE 7:**
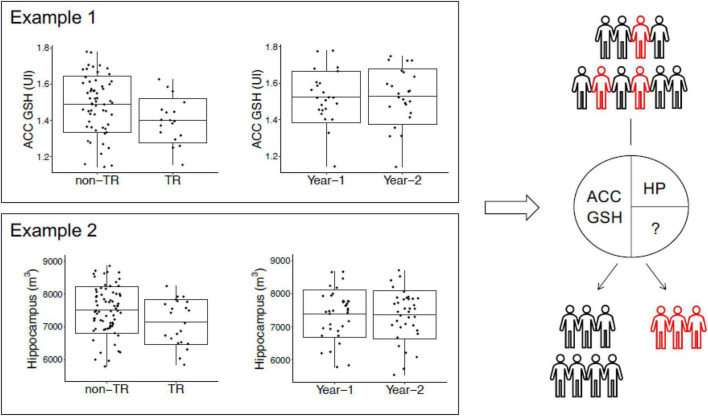
The cross-sectional study **(left)** observed reduced anterior cingulate cortex glutathione (GSH) levels and hippocampus volumes in non-responders; however, the longitudinal study data **(right)** found that anterior cingulate cortex GSH and hippocampus volume are stable over time in patients; these results imply that anterior cingulate cortex GSH and hippocampus volume may be trait markers for non-responders. Studying data from multimodal longitudinal cohorts may be a promising approach in the future to identify biomarkers for schizophrenia or a subset of schizophrenia patients. This figure shows anterior cingulate cortex GSH levels and the HP volume as potential biomarkers for TR to antipsychotics (Zhao et al., 2019; Picó-Pérez et al., 2022). Future studies are expected to identify additional biomarkers for TR and a combination of multimodal biomarkers that may be useful for the early diagnosis of TR in the clinic.

One meta-analysis noted that gray matter volume decreases in patients with schizophrenia are significant and in patients with long-term medication, the right STG/insula cluster showed the greatest diminution in volume. These changes occurred early in the disease but more frontal and temporal locations show gray matter volume declines with longer term disease. At the same time, those patients with treatment resistance psychosis show decreased hippocampal and superior frontal gyrus volumes, and glutathione levels in the anterior cingulate cortex, when compared to responders (Seethalakshmi et al., 2006; Figure 7).

A glutathione primacy model separates those patients with glutathione deficit from those without, and allows triage of patients into those benefiting from glutathione precursor medications (*N*-acetylcysteine) from those who may not benefit (Palaniyappan et al., 2021). Why has glutathione been highlighted? Glutathione is the primary antioxidant in the brain, and glutathione deficit can lead to injury to white matter, increased excitotoxicity, and oligodendrocyte cell death. The authors argue there may be a glutathione -deficit subtype of early-stage schizophrenia in which patients may maximally benefit from glutathione precursor medications (Palaniyappan et al., 2021).

#### Magnetoencephalography

Magnetoencephalography (MEG) is an electrophysiological measurement instrument which measures the magnetic fields emitted by postsynaptic neuronal activity of the brain in real time. MEG directly measures brain function with millisecond temporal resolution, unlike functional MRI which only measures the much slower delayed cerebral blood flow occurring in response to the neuronal activity, or MRI which shows brain structure. Because the skull and head soft-tissues are transparent to magnetic (but not electric) fields, the neuronal current sources can be localized with a few-millimeters of spatial resolution with MEG, significantly better than with electroencephalography. When fused with the patient’s brain anatomic structure shown on MRI, MEG can even show movies of the 3D location of brain activity (spontaneous or evoked) as it occurs in real-time, with millisecond temporal resolution (Hämäläinen et al., 2020).

Using MEG to evaluate patients with schizophrenia compared to normal controls provides useful information (a) in the resting state (measuring spontaneous brain rhythms); (b) in evaluating auditory evoked responses; and (c) in evaluating functional connectivity networks (Edgar et al., 2020).

The most robust finding of resting-state MEG in patients with schizophrenia is increased slow wave activity in delta (1–4 Hz) and theta (4–8 Hz) bands, which localize to frontotemporal and parietal regions in adults with schizophrenia (Edgar et al., 2020); but most studies only measure group differences of patients vs. healthy controls. However, if adequate normative databases are acquired, it appears that the distribution of MEG slow-waves can distinguish individual patients with schizophrenia from normal controls and other psychiatric diseases such as affective/neurotic disorders (Rockstroh et al., 2007).

Other resting-state frequency bands in patients with schizophrenia studied with MEG include the alpha (9–12 Hz) and beta (13–30 Hz) bands. For example, Zeev-Wolf et al. (2018) found that patients high in positive symptoms had widespread low alpha power; and patients high in negative symptoms showed greater beta power in the left hemisphere than those patients not dominated by negative symptoms.

Gamma-band (30–71 Hz) activity in subjects with schizophrenia has also been studied with MEG. As one example, Kissler et al. (2000) studied gamma power distribution during rest, and during a mental arithmetic task. Normal subjects doing arithmetic showed increased gamma power over the left frontal and frontotemporal regions, whereas patients with schizophrenia lacked this increase, or showed increase in the contralateral right hemisphere. Also, the distribution of resting-state gamma activity differed between healthy controls and patients with schizophrenia.

Lottman et al. conducted a study on 19 patients with schizophrenia and 24 healthy subjects using resting-state 7 Tesla fMRI and MEG. They found a decreased connectivity between task positive networks and sensorimotor in the delta frequency band in MEG (Lottman et al., 2019).

Because auditory hallucinations are a prominent manifestation of schizophrenia, many electroencephalography and MEG studies of auditory encoding have been performed, examining activity in the superior temporal gyri, including time-frequency plots of total power, and inter-trial coherence or phase-locking plots. Chen et al. (2019) found that control subjects had stronger auditory encoding activity in superior temporal gyri than patients with schizophrenia, and showed that unaffected relatives of the patients had responses which were similar to control patients in the superior temporal gyri, but were abnormal and similar to the patients, in the left superior frontal gyrus.

Some but not all MEG studies show abnormal auditory gating in patients with schizophrenia: presented with two clicks 500 ms apart, normal subjects show decreased auditory activation response to the second click compared to the first, indicating habituation to the stimulus. Patients with schizophrenia do not show such decreased response to the second click (Edgar et al., 2020).

Because of MEG’s ability to sensitively measure neural oscillatory processes, and its good spatial localization using advanced source-reconstruction algorithms, MEG is a useful technique to evaluate whole-brain functional activity in normal subjects and patients with schizophrenia, superimposing the MEG neural activity on the brain MRI images. Both increased and decreased functional connectivity has been observed in patients with schizophrenia vs. controls, in resting state and during various tasks (Edgar et al., 2020).

For example, Hirvonen et al. (2017) found that both local and cross-region gamma-band (30–120 Hz) MEG synchronization was lower in patients with schizophrenia than normal controls performing a perceptual integration task. Alamian et al. (2017) have systematically reviewed the literature of resting-state MEG networks in schizophrenia, and described the strengths and methodological challenges of the technology.

## Imaging changes in different manifestations of schizophrenia

As described above in multiple sections, the pattern of imaging findings may vary depending upon whether the patients’ symptoms are negative (decreased motivation and diminished expressiveness) or positive (delusions, hallucinations, and aggressive features). Thus, of the five types of schizophrenia described in DSM-4, three (catatonic, residual, and disorganized schizophrenia) are more negative in expression. Paranoid and undifferentiated schizophrenia types are more positive in symptomatology. Negative symptom schizophrenia is associated with lower FDG PET uptake, lower perfusion and lower activation on fMRI, particularly in cortical and subcortical regions (Rahmim and Zaidi, 2008; Jales and Santos-Filho, 2021) whereas positive symptoms equate to greater activation, uptake and perfusion in temperolimbic and deep gray matter structures (Jales and Santos-Filho, 2021).

Zhang et al. (2015) uncovered structural brain changes in different subtypes of schizophrenia using MRI pattern analysis. They found that patients with predominantly negative symptoms had a significant decrease in cerebral volume compared to patients with predominant disorganized and predominant positive symptoms. Also, in comparison with predominant positive and predominant disorganized symptoms, predominant negative symptoms were inversely associated with cerebellar size. Mega et al. (1997) found a greater decrease of GM volume in patients with predominant positive symptoms in the orbitofrontal cortex, insular cortex and temporal pole compared to those of predominant disorganized. Negative symptoms are associated with decreases in the volume of ventromedial prefrontal cortex, thalamus and orbitofrontal cortex volume (Andreasen et al., 1998). The patients with predominant positive symptoms were shown to have dramatically reduced gray matter volume in the ventromedial prefrontal cortex (Zhang et al., 2015). Two studies (Xiao et al., 2015) found a significant decrease in gray matter volume in inferior frontal, left orbitofrontal, and medial frontal gyri in paranoid/positive symptom schizophrenia.

Two voxel-based morphometry correlation studies (Venkatasubramanian, 2010; Bergé et al., 2011) found a negative correlation between cerebellar volume and Positive and Negative Syndrome Scale negative scores. The neurologic soft signs (deficits in sensory integration, motor coordination, and sequencing of complex motor acts) also correlate with decreased cerebral volume (Taylor et al., 2012). Martí-Bonmatí et al. (2007) also reported a correlation between (1) hallucinations and reduced inferior frontal and orbitofrontal cortex volume as well as (2) aggressive behaviors and a decrease in ventromedial prefrontal cortex volume in patients with schizophrenia.

As stated previously, positive and negative symptoms may affect MEG results [patients high in positive symptoms have widespread low alpha power while patients high in negative symptoms showed greater beta power in the left hemisphere (Zeev-Wolf et al., 2018)], PET results [negative symptoms are linked to lower FDG-PET metabolism in all regions of interest in frontal, parietal, temporal, limbic, thalamic and occipital regions (Tamminga et al., 1992)], rs-fMRI findings [positive symptoms are correlated with the default mode network, central executive, and salience networks’ dysconnectivity (Rotarska-Jagiela et al., 2010; Woodward et al., 2011; Khadka et al., 2013; Palaniyappan et al., 2013)], and MRS metabolite values [higher glutamate to creatine ratio in the medial frontal cortex was positively associated with positive schizophrenia symptomatology severity (Merritt et al., 2021)].

Many studies reported a significant decrease in left and right asymmetry in the white matter tracts which are responsible for language processing (arcuate fasciculus) in patients with schizophrenia (Falkenberg et al., 2020). Bilateral asymmetry also has been reported (Ćurčić-Blake et al., 2015). In a recent study, Beresniewicz et al. explored changes in white matter (e.g., corticospinal, frontotemporal, anterior thalamus tracts, cingulum, and inferior fronto-occipital fasciculus) and their relation with hallucination in patients with schizophrenia, using MR DTI. They found a greater decrease in fractional anisotropy in patients with hallucination compared to patients without this symptom (Beresniewicz et al., 2021).

Geisler et al. (2015) categorized patients with schizophrenia into four cognitive clusters and provided their correlation with brain structures and functions (see Table 2).

**TABLE 2:** Cognitive clusters in schizophrenia relate to cognitive deficits, neural anatomy, and function as measured on fMRI.

Categorizations	Symptoms	Neurological impairments (fMRI)
Cluster 1	Low processing speed and verbal fluency	Cortical thickness, reduced neural size, dendrite trees, interneural neuropil, synaptic spines, and cortical afferents
Cluster 2	Impaired motor control and verbal memory	Reduced neural activity in he left precentral gyrus, Broca, reduced right hemispheric hippocampal volume
Cluster 3	Impaired face memory, signal detection and processing	Cortical thinning in the adjacent areas, lingual gyrus and middle temporal gyrus Increased neural response in temporal regions during working-memory tasks
Cluster 4	Impaired cognitive functions, high positive symptoms	Generalized cortical thinning Significant widening in bilateral sulci

The clusters are generally classified as follows: Cluster 1: severe and profound global dysfunction; Cluster 2: near normative performance with mild dysfunction in verbal memory; Cluster 3: moderate-severe with more prominent executive than memory dysfunction; and Cluster 4: moderate-severe with more prominent memory than executive dysfunction.

Xie et al. presented brain changes in deficit schizophrenia patients using MRI. They compared cortical thickness and surface area in 33 deficit schizophrenia, 41 non-deficit schizophrenia, and 41 healthy subjects. They discovered convergent cortical thinning in the bilateral inferior frontal gyri and left superior temporal gyrus. In addition, impaired cognition and visuospatial memory deficit was positively related to right inferior frontal cortex in deficit schizophrenia and non-deficit schizophrenia patients. However, deficit schizophrenia patients showed more cortical thinning especially in left temporo-parietal junction area compared to non-deficit schizophrenia group. Surface area in all three groups showed no specific differences (Xie et al., 2019). In another study conducted by Takahashi et al., the interthalamic adhesion, cavum septi pellucidi, and surface morphology were evaluated on 38 deficit schizophrenia patients, 37 non-deficit schizophrenia patients, and 59 controls. They found that deficit schizophrenia group exhibited a shorter interthalamic adhesion, compared to non-deficit schizophrenia and healthy subjects. In addition, deficit schizophrenia group showed different distribution of orbitofrontal cortex sulcogyral pattern and fewer posterior orbital sulci compared to healthy subjects (Takahashi et al., 2017).

## Effect of therapies

### Cognitive therapy

#### Cognitive remediation therapy

There are many types of therapies, either psychological or pharmacological, available for schizophrenia. Cognitive remediation therapy is considered one of the psychological treatments in schizophrenia. Cognitive remediation therapy uses drill and practice, compensatory, and adaptive strategies to improve deficits in memory, attention, and problem solving (Kanahara et al., 2022). Therapy is aimed at helping individuals with schizophrenia that experience cognitive impairment. Wykes et al. (2002) investigated the effect of cognitive remediation therapy in schizophrenia patients and its association with brain activity alteration before and after treatment. In this study, fMRI analysis showed a significant increase in activation in regions that correlate with memory after successful cognitive remediation therapy treatment, such as increased activity in bilateral occipital and right inferior frontal gyrus. Bor et al. (2011) detected a significant increase in the activity of left cingulate gyrus, left inferior or middle frontal gyrus and left inferior parietal lobule in patients after cognitive remediation therapy while performing a working memory task-based fMRI experiment. Haut et al. (2010) discovered that patients who undergo cognitive remediation therapy showed a large increase in the activity of anterior cingulate gyrus, dorsolateral prefrontal cortex, and frontopolar cortex while engaged in a verbal and visual working memory task. Subramaniam et al. (2012) showed that patients after cognitive remediation therapy have a notable increase in the activity of medial prefrontal cortex during a reality monitoring task. Vianin et al. (2014) also found an increased activity in Broca’s area utilizing a verbal fluency task (left inferior frontal gyrus, left precentral gyrus, bilateral inferior parietal lobule, precentral gyrus, and middle occipital gyrus). In addition, Penadés et al. (2013) found that patients on therapy showed a rise in fractional anisotropy index in the anterior part of the genu of the corpus callosum, implying better integrity of white matter tracts.

#### Cognitive behavioral therapy

Cognitive behavioral therapy for neuropsychiatric diseases includes psychosocial education about the illness, goal setting, symptom monitoring, cognitive restructuring, skills training, safety monitoring, coping techniques, and graded homework assignments. While cognitive remediation therapy helps improve the underlying neuropsychological functions of cognition, concentration and learning, cognitive behavioral therapy teaches the patient how to think through emotionally challenging problems. Kumari et al. (2011) found that the fMRI findings of patients who underwent cognitive behavioral therapy had decreased in activity of thalamus, frontal, putamen, insula and occipital areas when performing an affect processing task (presentation of black and white photographs depicting facial emotions). Mason et al. (2016) also used a facial emotion task and showed that psychotic patients had greater amygdala connectivity with the insula and visual areas and less connectivity with somatosensory areas at baseline, which corrected after cognitive behavioral therapy with increases in amygdala connectivity with dorsolateral prefrontal cortex and inferior parietal lobule. Positive symptom improvement correlated best with improved amygdala-inferior parietal lobule connectivity. They concluded that cognitive behavioral therapy reorganized amygdala prefrontal cortex connections that mediate the hyper-reactivity of patients with schizophrenia (i.e., paranoia) from social threats.

### Antipsychotic medications

Kani et al. (2017) showed increased functional activation in the superior frontal gyrus middle frontal gyrus and inferior frontal gyrus using fMRI after antipsychotic medication treatment. On MR spectroscopy, the antipsychotic dose was also negatively associated with glutamate and glutamine levels in the medial frontal cortex (estimate, 0.10 reduction per 100 mg chlorpromazine) (Merritt et al., 2021).

van Veelen et al. (2011) found no significant change in dorsolateral prefrontal cortex activation before and after administration of second-generation antipsychotics using a Sternberg working memory task in healthy controls and those receiving treatment. However, at baseline, those patients with schizophrenia who did NOT respond to the drugs had a lower level of dorsolateral prefrontal cortex activation on the task—it was predictive of treatment response. Ikuta et al. (2014) evaluated a link between risperidone and aripiprazole treatment on intentional control during a multi-source interference task on fMRI. They found a direct link between reduced right globus pallidus activity and improved and accurate responses as well as a decrease in thought disturbance. Keedy et al. (2009) also found an increase in bilateral supplementary eye fields, left frontal eye fields, and bilateral cerebellum functional activity during visual attention and sensorimotor tasks and a decrease activity in other distributed brain regions in patients treated with risperidone, ziprasidone, or haloperidol.

Miyazawa et al. (2022) noted that that clozapine prolongs cortical silent period (the interruption of voluntary muscle contraction following a motor-evoked potential triggered by transcranial magnetic stimulation over the primary motor cortex) due to increase GABA transmission with GABA_B_ receptors.

Sarpal et al. (2015) examined the association between antipsychotic treatment (e.g., aripiprazole or risperidone) and activity changes in striatal areas of the brain in patient with schizophrenia via resting-state fMRI. They found that after treatment, patients had improved functional connectivity among right dorsal caudate and a number of prefrontal areas such as the anterior cingulate, right dorsolateral prefrontal cortex, and orbitofrontal cortex. In addition, with the improvement in symptoms, the right ventral nucleus, caudate, nucleus accumbens and hippocampus showed a significant increased functional connectivity. The same held true for the anterior insula and ventral putamen’s seed regions. On the other hand, as psychotic symptoms got worse, there was a significant decrease in functional connectivity between ventral caudate and posterior areas.

### Transcranial magnetic stimulation

#### Repetitive transcranial magnetic stimulation

Repetitive transcranial magnetic stimulation is a non-invasive procedure which is used to treat mental disorders. Fitzgerald et al. (2007) found that patients with schizophrenia who have undergone TMS treatment showed a significant increase in the activity of left precentral gyrus, left temporoparietal regions, and left inferior frontal gyrus. Kindler et al. (2013) also found a decrease in cerebral blood flow in the left primary auditory cortex, cingulate gyrus and Broca’s region after repetitive transcranial magnetic stimulation via pseudocontinuous magnetic resonance-arterial spin labeling in their study of 30 patients with schizophrenia.

#### Transcranial direct current stimulation

Transcranial direct current stimulation is a non-invasive, painless brain stimulation treatment variant of transcranial magnetic stimulation. Mondino et al. (2016) found that transcranial direct current stimulation can decrease negative symptoms course and the severity of auditory verbal hallucination in patients with schizophrenia. This improvement was associated with reduction in functional connectivity between the left anterior insula and left temporoparietal junction (middle and superior temporal gyri and Wernicke’s area). Palm et al. (2016) also found a great increase in resting state functional connectivity between left inferior or middle temporal gyrus and left dorsolateral prefrontal cortex, right insula and right dorsolateral prefrontal cortex, right thalamus and right subgenual gray matter with transcranial direct current stimulation treatment.

## Age, gender, and comorbidity effects

Nopoulos et al. (1997) assessed the association between sex and structural brain abnormalities in schizophrenia. MRI and postmortem research showed reduced total tissue and increased total ventricular volume in men with schizophrenia but there was only a slight increase in overall ventricular volume in women with schizophrenia compared to healthy controls. Also, men had smaller medial temporal and frontal volumes. Moreover, men with schizophrenia have much smaller left planum temporale, left superior temporal gyrus, left Heschel’s gyrus and left hippocampal volumes compared to women. In addition, greater sulcal volume and reduced thalamic size are seen more in male patients (Nopoulos et al., 1997).

According to Jones et al. (2006) age is an underlying factor in patients with schizophrenia when assessing water diffusivity in the fronto-temporal pathway. They found that as people with schizophrenia age, there was a significant increase in fractional anisotropy and decrease in mean diffusivity. As a result, white matter maturational alterations are delayed in patients with schizophrenia. Therefore, in order to perform diffusion tensor MRI studies in schizophrenia, the age of patients and age of onset of symptoms should be considered (Jones et al., 2006). Mathalon et al. (2001) noted a decrease in fronto-temporal gray matter volume with age in schizophrenia. van Haren et al. (2008) found that in patients with schizophrenia before the age of 45, there was an increase in cortical gray matter atrophy. Raz et al. (2005) found a decrease in white matter integrity which was increased in older patients with schizophrenia. Finally, Rosenberger et al. (2008) found a great decrease in fractional anisotropy in the uncinate and cingulum with age in their subjects with schizophrenia. They stated that, by comparison, there is no association between decreased functional anisotropy and age in the healthy population.

There are many diseases and factors that should be considered in assessing and imaging schizophrenia. Addiction is very prevalent among patients with schizophrenia. Schiffer et al. (2010) found that there is an association between substance abuse and increased impulsivity. They found that gray matter volume deficits are seen in patient who have schizophrenia with and without substance abuse, but these deficits were much larger in addicted ones (nearly 8%). Although there is a known association between schizophrenia and decreased gray matter volume in lateral orbitofrontal and temporal areas, the medial orbitofrontal, frontotemporal cortex and anterior cingulate volumes are independently decreased with addiction.

## Pitfalls

Although there are numerous imaging studies in patients with schizophrenia, most of the cohorts lack appropriate balancing for age, sex, comorbidities, and therapeutic pharmacologic interventions. Controlling for cigarette smoking, marijuana use, illicit drug habits, and educational level are important to a clearer understanding of this patient population. It has been shown in schizophrenia and other disorders that the patterns of brain activation on resting state and task-based fMRI in patients with neuropsychological disorders change with medication use. Additionally, the duration of disease appears to affect the neurophysiology of schizophrenia as evidenced by response to therapy studies that show such impact. The symptom complexes of patients with schizophrenia are also variable and affect imaging findings (see above). It stands to reason that DTI or fMRI or PET patterns of brain function and structure may vary depending upon whether the patient is experiencing hallucinations (positive symptoms) versus abulia (negative symptoms). Our review of the literature is limited by a great heterogeneity in neuroimaging data, depending on the diagnoses, sample sizes, tasks or paradigms used. Additionally, patients with schizophrenia are not immune to other psychiatric disorders such as autism, bipolar disorder, attention deficit hyperactivity disorder, and depression. Even general medical disorders such as diabetes, hypertension, and hyperlipidemia, which may affect vascular reactivity and perfusion in the brain, are rarely controlled for in neuroimaging studies of patients with schizophrenia.

## Future considerations

Because of the limitations described above, the neuroradiology medical literature would benefit from larger scale studies of individuals with new onset psychosis meeting DSM-5 criteria for schizophrenia. Sharing data from multiple groups (as in ENIGMA) to acquire more homogeneous study groups would be a useful effort. These patients should be screened for other medical and psychological comorbidities to ensure a homogeneous patient population. Ideally the patients will not be receiving medications at the initial evaluation of their brain. To maximize the benefit, a tailored structural and functional MRI protocol that examines diffusion tensor imaging, resting state and standardized task-based fMRI, MR spectroscopy, perfusion imaging, as well as high resolution volumetric MRI sequences that include quantitative susceptibility weighted imaging (to can assess for iron content in basal ganglionic structures and overall brain iron content) would be most useful. After a baseline scan, treatment could be initiated in a standardized fashion so as to determine how anti-psychotic medications, TMS, and/or behavioral therapy impacts the brain functionally and structurally. Serial scanning to assess the role of the duration of disease would also be valuable to the literature.

## Conclusion

The neuroimaging studies to date on schizophrenia have yielded many observations about the brain in patients. Structurally there is consensus that there is gray matter volume loss and ventricular enlargement in patients with schizophrenia. Both structural studies (showing decreased volume) and functional MR studies (showing hypoactivation and/or reduced connectivity) have focused on (1) thalamocortical connections, (2) amygdalostriate pathways, (3) cortico-striatal-limbic hubs, (4) default mode network, and (5) salience networks. Diffusion weighted images and DTI derived indices showed widespread abnormalities in the white matter of patients with schizophrenia. FDG PET has consistently shown hypofrontality in brain uptake. However, this may be mitigated in part by the dominance of positive or negative symptoms. Dopamine PET studies have shown increased dopamine synthesis capacity in the basal ganglia while cannabinoid receptor studies have mainly focused on increased receptor concentration in the nucleus accumbens. Lower levels of *N*-acetylaspartate and glutamate on MRS have been demonstrated most consistently in the anterior cingulate cortex in patients with schizophrenia. Other MRS investigators have focused on glutathione, usually reduced in patients with schizophrenia, to predict whether the patients will respond to treatment. These treatments, be they cognitive therapies, anti-psychotic medications, or direct stimulation magnetic techniques, may affect the structure and function of the brains of patients with schizophrenia. Finally, we note that the multitude of variables around patient demographics, disease types and manifestations, duration of disease, therapeutic state, comorbidities, and coexistent psychiatric and/or substance abuse issues in patients with schizophrenia has prevented a definitive picture of the morphologic and functional pathophysiology of this complex disorder.

## Author contributions

MD and DY: conception or design of the work. MD, FD, KY, PB, RL, and DY: acquisition, analysis, or interpretation of data for the work, drafting the work, or revising it critically for important intellectual content, final approval of the version to be published, agreed to be accountable for all aspects of the work in ensuring that questions related to the accuracy or integrity of any part of the work were appropriately investigated and resolved, they had full access to all of the data in this study, and take complete responsibility for the integrity of the data, and the accuracy of the data analysis.
